# The re-emergence of highly pathogenic avian influenza H7N9 viruses in humans in mainland China, 2019

**DOI:** 10.2807/1560-7917.ES.2019.24.21.1900273

**Published:** 2019-05-23

**Authors:** Deshan Yu, Guofeng Xiang, Wenfei Zhu, Xia Lei, Baodi Li, Yao Meng, Lei Yang, Hongyan Jiao, Xiyan Li, Weijuan Huang, Hejiang Wei, Yanping Zhang, Yan Hai, Hui Zhang, Hua Yue, Shumei Zou, Xiang Zhao, Chao Li, Deng Ao, Ye Zhang, Minju Tan, Jia Liu, Xuemei Zhang, George F. Gao, Lei Meng, Dayan Wang

**Affiliations:** 1 These authors contributed equally in this study as first authors; 2 Gansu Provincial Center for Disease Control and Prevention, Lanzhou China; 3 Jiuquan Center for Disease Control and Prevention, Jiuquan, China; 4 National Institute for Viral Disease Control and Prevention, Chinese Center for Disease Control and Prevention; WHO Collaborating Center for Reference and Research on Influenza; Key Laboratory for Medical Virology, National Health Commission, Beijing, China; 5 Inner Mongolia Center for Disease Control and Prevention, Hohehot, China; 6 Alasan League Center for Disease Control and Prevention, Alasan, China; 7 Chinese Center for Disease Control and Prevention, Beijing, China; 8 These authors contributed equally as last authors in this study

**Keywords:** Highly pathogenic avian influenza virus, A(H7N9), Phylogenetic analysis, Human case

## Abstract

After no reported human cases of highly pathogenic avian influenza (HPAI) H7N9 for over a year, a case with severe disease occurred in late March 2019. Among HPAI H7N9 viral sequences, those recovered from the case and from environmental samples of a poultry slaughtering stall near their home formed a distinct clade from 2017 viral sequences. Several mutations possibly associated to antigenic drift occurred in the haemagglutinin gene, potentially warranting update of H7N9 vaccine strains.

Since March 2013, influenza A(H7N9) viruses have caused five epidemic waves of zoonotic infections with a large number of reported human cases (1,567 in total up to February 2018). The first wave lasted until September 2013, and the following four occurred annually between October and September of the next year from 2013/14 to 2016/17. During the fifth wave in 2016/17, the emergence of highly pathogenic avian influenza (HPAI) H7N9 viruses raised wide global concern [1]. Compared to low pathogenic avian influenza (LPAI) A(H7N9) viruses, HPAI H7N9 viruses maintained the capacity to bind both human and avian receptors [2] and unreduced transmissibility in mammalian animal models, but exhibited higher virulence and broader tissue tropism [3-5]. Subsequent to 31 human HPAI H7N9 cases being reported in China in the fifth wave, their numbers decreased dramatically from October 2017, with only one additional HPAI H7N9 human case up to February 2018. These 32 latest human cases covered nine provinces of China. During the following 14 months, neither LPAI H7N9 nor HPAI H7N9 was reported in humans in the country. Several HPAI H7N9 outbreaks occurred in poultry, with the latest in March 2019 in peacocks in Liaoning province (http://www.moa.gov.cn/).

In late March 2019, a person in Inner Mongolia, China, presenting with severe pneumonia and respiratory failure was confirmed with HPAI H7N9. The re-emergence of a human HPAI H7N9 virus infection after reports of such cases had ceased for more than a year caused high public health concerns. We hereby describe this case and analyse genome features of the viruses causing the infection and of viruses found near the case’s residence.

## Case description

The patient, a person in their early 80s with underlying cardiovascular disease, lived in the Inner Mongolia Autonomous region. The first symptoms (day 1 of illness) occurred at the end of March 2019 and included chills, cough, fever (39.0 °C), headache, muscular soreness and shortness of breath. On day 6 of illness, the patient was admitted to a local hospital. Acute heart failure, hypertension, pneumonia, residuals of cerebral infarction and venous thrombosis were diagnosed. On day 7, the clinical condition deteriorated markedly and the patient was transferred to a hospital in Gansu province, a province near Inner Mongolia. Based on clinical signs and computed tomography (CT) results, bilateral pneumonia and emphysema pulmonum were diagnosed. A patient’s throat swab sampled in the beginning of April was positive for influenza A(H7N9) viruses. On day 19, the patient died due to secondary bacterial infections and development of multiple organ failure.

## Environmental investigations

In China, regular passive surveillance of poultry related environments (including live poultry markets) has been conducted every year since 2008, by local Centers for Disease Control and Prevention (CDC). Influenza positive specimens are sent to the Chinese National Influenza Centre, Institute for Viral Disease Control and Prevention (IVDC), China CDC, for virus isolation.

Concerning the Alashan League in the Inner Mongolia Autonomous region where the patient lived, 50 to 70 environmental samples are collected annually. In 2018, all 50 such environmental samples were found to be negative for influenza A(H7N9) viruses. Upon the identification of the case, active surveillance was conducted. As there were two live poultry slaughtering stalls at 200 metres from the case’s home, a total of 51 samples were obtained from both stalls. Of these, 22 H7N9 positive samples were detected, all exclusively originating from the same stall. Poultry vaccination had been adopted in the region, however, investigations revealed that the particular poultry from the H7N9 positive stall had not been vaccinated.

## Sequencing and identity analysis of nt sequences

Respiratory samples had been collected from the patient on day 8, 10 and 11 of illness. Real-time reverse transcription (RT)-PCR was performed and A(H7N9) positive samples were propagated in the allantoic cavity of 9–10 days old specific pathogen free (SPF) embryonated chicken eggs for 48h–72h at 37 °C in biosafety level 3 laboratory. Five virus strains were isolated from throat swab or lower respiratory tract samples, and termed as A/Gansu/23276/2019 (GS23276, H7N9), A/Gansu/23275/2019 (H7N9), A/Gansu/23277/2019 (H7N9), A/Gansu/23447/2019 (H7N9), A/Gansu/23453/2019 (H7N9).

For the 22 H7N9 positive environmental samples, six viruses were isolated.

In order to achieve full genome sequencing of the viruses, RNA was extracted from the original samples or isolated viruses and subjected to RT and amplification. Whole genome sequencing was implemented on the MiSeq high-throughput sequencing platform (Illumina, Inc. San Diego, California (CA)). Data analysis and genome sequences acquisition were conducted according to a previous study [6]. Full genome sequences were obtained from three original clinical samples and two original environmental samples, as well as five human isolates and six environmental isolates. The sequences were submitted to Global Initiative on Sharing All Influenza Data (GISAID) [7] with the accession number of EPI1431481–EPI1431608.

The nt sequences of the H7N9 viruses in this study shared 99.9% to 100% identity in each of the eight genes of the influenza virus genome, suggesting that the H7N9 viruses in this study possess a similar genetic constellation and belong to a common evolutionary lineage. The Basic Local Alignment Search Tool (BLAST) results of the full genome nt sequences found that all eight genes of GS23276 shared the highest identity with HPAI H7N9 viruses isolated in 2017, with varied identity between 97.9% to 99.1%.

## Evolutionary analyses

**Figure 1:**
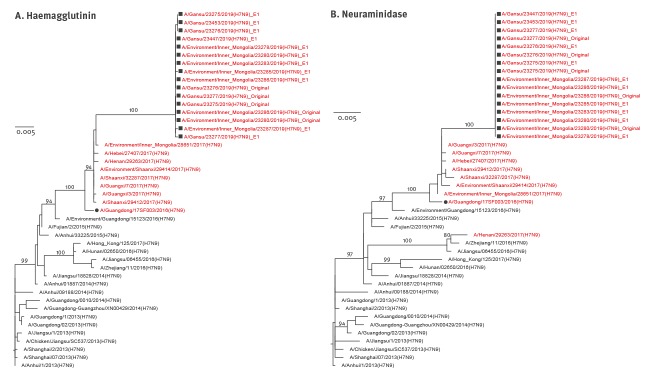
Phylogenetic analyses of the (A) haemagglutinin and (B) neuraminidase gene segments of HPAI H7N9 viruses recovered from an infected patient and from environmental samples collected nearby, Inner Mongolia Autonomous region, China, April 2019

HPAI: highly pathogenic avian influenza; MEGA: Molecular Evolutionary Genetics Analysis; MUSCLE: MUltiple Sequence Comparison by Log- Expectation.

In order to analyse the relationship of these HPAI H7N9 viruses genetically, viral sequences representing prototypes of each of the waves of H7N9 outbreaks since the start of the outbreaks in 2013, as well as sequences with highest identity, were downloaded from GISAID, to generate a maximum likelihood tree using Molecular Evolutionary Genetics Analysis (MEGA) software version 7. Phylogenetic analyses of the haemagglutinin (HA) genes showed that all the HPAI H7N9 viruses fall into one cluster, with the candidate vaccine strain A/Guangdong/17SF003/2016 (GD/SF003, H7N9) (Figure 1A). All human and environmental HPAI H7N9 isolates from this study grouped into a single subclade, which showed a relatively long genetic distance to other HPAI H7N9 viruses. These results indicate that the re-emerged HPAI H7N9 viruses in this study may have originated from GD/SF003-like viruses but are divergent from the closest known ones before 2019. 

Multiple sequence alignments were performed with the MUSCLE programme using MEGA software version 7. Maximum likelihood trees on (A) haemagglutinin and (B) neuraminidase genes were conducted using the general time reversible + Γ nt substitution model with 1,000 bootstraps, respectively. HPAI H7N9 viruses are highlighted in red. The investigated viruses and the candidate HPAI H7N9 vaccine strain are shown with a solid square and circle, respectively. Bootstrap values higher than 60 are shown. Horizontal distances are proportional to genetic distance, as shown by the scale bars.

**Figure 2:**
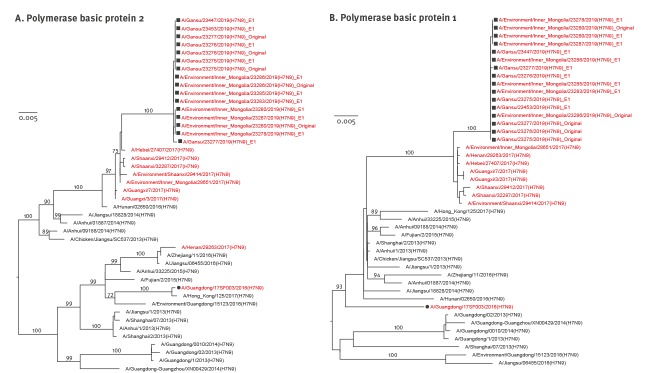
Phylogenetic analyses of the (A) polymerase basic protein 2 and (B) polymerase basic protein 1 gene segments of HPAI H7N9 viruses recovered from an infected patient and from environmental samples collected nearby, Inner Mongolia Autonomous region, China, April 2019

HPAI: highly pathogenic avian influenza; MEGA: Molecular Evolutionary Genetics Analysis; MUSCLE: MUltiple Sequence Comparison by Log- Expectation.

**Figure 3:**
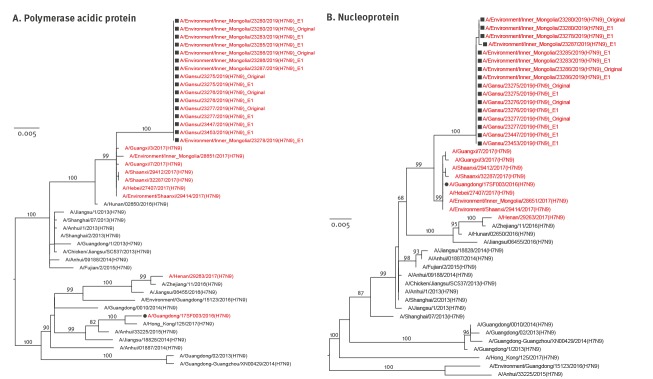
Phylogenetic analyses of the (A) polymerase acidic protein and (B) nucleoprotein gene segments of HPAI H7N9 viruses recovered from an infected patient and from environmental samples collected nearby, Inner Mongolia Autonomous region, China, April 2019

HPAI: highly pathogenic avian influenza; MEGA: Molecular Evolutionary Genetics Analysis; MUSCLE: MUltiple Sequence Comparison by Log- Expectation.

**Figure 4:**
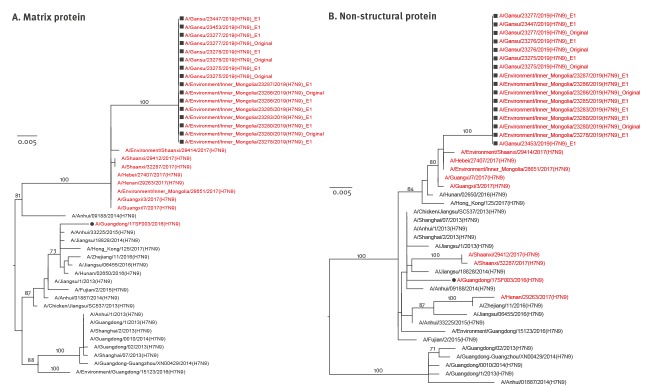
Phylogenetic analyses of the (A) matrix protein and (B) non-structural protein gene segments of HPAI H7N9 viruses recovered from an infected patient and from environmental samples collected nearby, Inner Mongolia Autonomous region, China, April 2019

HPAI: highly pathogenic avian influenza; MEGA: Molecular Evolutionary Genetics Analysis; MUSCLE: MUltiple Sequence Comparison by Log- Expectation.

Like HA genes, the other seven genes showed the same evolutionary pattern (Figure 1B, Figure 2, Figure 3, Figure 4). All eight gene segments of these re-emerged HPAI H7N9 isolates belonged to the same group and no re-assortment was observed.

Multiple sequence alignments and maximum likelihood trees were conducted as for Figure 1 on the (A) polymerase basic protein 2 and (B) polymerase basic protein 1 genes. HPAI H7N9 viruses are highlighted in red. The investigated viruses and the candidate HPAI H7N9 vaccine strain are shown with a solid square and circle, respectively. Bootstrap values higher than 60 are shown. Horizontal distances are proportional to genetic distance, as shown by the scale bars.

Multiple sequence alignments and maximum likelihood trees were conducted as for Figure 1 on the (A) polymerase acidic protein and (B) nucleoprotein genes. HPAI H7N9 viruses are highlighted in red. The investigated viruses and the candidate HPAI H7N9 vaccine strain are shown with a solid square and circle, respectively. Bootstrap values higher than 60 are shown. Horizontal distances are proportional to genetic distance, as shown by the scale bars.

Multiple sequence alignments and maximum likelihood trees were conducted as for Figure 1 on the (A) matrix protein and (B) non-structural protein genes. HPAI H7N9 viruses are highlighted in red. The investigated viruses and the candidate HPAI H7N9 vaccine strain are shown with a solid square and circle, respectively. Bootstrap values higher than 60 are shown. Horizontal distances are proportional to genetic distance, as shown by the scale bars.

## Key molecular marker analyses

**Figure 5:**
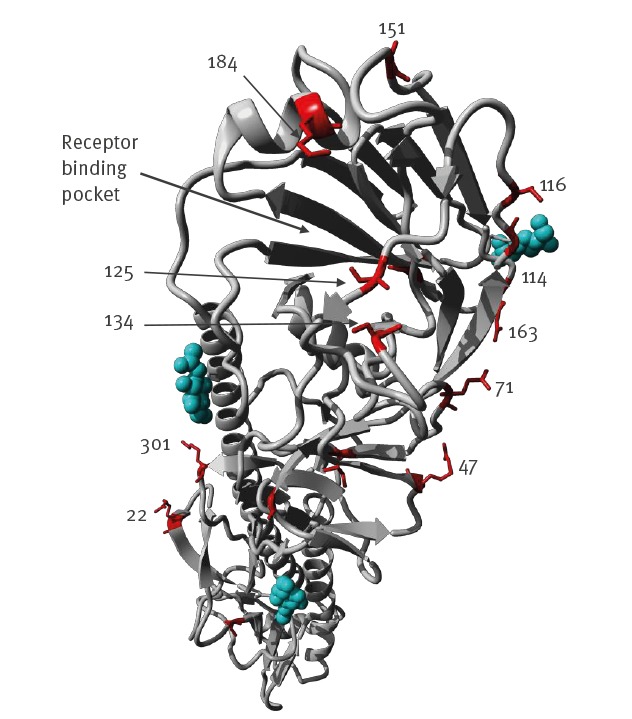
Structural view of mutations in the haemagglutinin 1 viral sequences recovered from a highly pathogenic avian influenza A(H7N9) infected patient and from environmental samples collected nearby, compared to candidate vaccine strain A/Guangdong/17SF003/2016(H7N9), Inner Mongolia Autonomous region, China, April 2019

The key molecular features associated with increased virulence in mammals, mammalian transmissibility, or antiviral resistance were further determined. All HPAI H7N9 viruses identified in this study contained a multiple basic amino acid motif (PEVPKRKRTAR↓G) at the HA gene cleavage site, which is identical to the cleavage site of the candidate vaccine strain GD/SF003, and indicates high pathogenicity in poultry. Comparison of the HA1 proteins of these viruses with the GD/SF003 led to identify 15 substitutions (Table 1 and Figure 5). Among them, R47K, G114R, V125T/A, and S134P (H7 numbering) were the previously reported immune escape mutations [8,9], indicating the possible antigenic variation of the re-emerged HPAI H7N9 viruses. 

**Table 1:** Mutations in haemagglutinin 1 of viral sequences recovered from an infected patient and from environmental samples collected nearby, compared to candidate vaccine strain A/Guangdong/17SF003/2016(H7N9), Inner Mongolia Autonomous region, China, April 2019^a^

Strain name	Passage	Sites number (H7 numbering)														
		9	22	47^b^	71	78	114^b^	116	125^b^	134^b^	151	163	169	184	261	301
A/Guangdong/17SF003/2016(H7N9)	E1	A	R	R	E	I	G	T	V	S	A	K	I	K	R	K
A/Gansu/23277/2019(H7N9)	Original	S	K	K	K	V	R	K	T	P	T	R	V	R	G	R
A/Gansu/23276/2019(H7N9)	Original	S	K	K	K	V	R	K	A/T	P	T	R	V	R	G	R
A/Gansu/23275/2019(H7N9)	Original	S	K	K	K	V	R	K	A/T	P	T	R	V	R	G	R
A/Gansu/23277/2019(H7N9)	E1	S	K	K	K	V	R	E	T	P	T	R	V	R	G	R
A/Gansu/23276/2019(H7N9)	E1	S	K	K	K	V	R	K	A	P	T	R	V	R	G	R
A/Gansu/23275/2019(H7N9)	E1	S	K	K	K	V	R	K	A	P	T	R	V	R	G	R
A/Gansu/23447/2019(H7N9)	E1	S	K	K	K	V	R	K	T	P	T	R	V	R	G	R
A/Gansu/23453/2019(H7N9)	E1	S	K	K	K	V	R	K	A	P	T	R	V	R	G	R
A/Environment/Inner Mongolia/23280/2019(H7N9)	Original	S	K	K	K	V	R	K	T	P	T	R	V	R	G	R
A/Environment/Inner Mongolia/23286/2019(H7N9)	Original	S	K	K	K	V	R	K	T	P	T	R	V	R	G	R
A/Environment/Inner Mongolia/23287/2019(H7N9)	E1	S	K	K	K	V	R	K	T	P	T	R	V	R	G	R
A/Environment/Inner Mongolia/23286/2019(H7N9)	E1	S	K	K	K	V	R	K	T	P	T	R	V	R	G	R
A/Environment/Inner Mongolia/23285/2019(H7N9)	E1	S	K	K	K	V	R	K	T	P	T	R	V	R	G	R
A/Environment/Inner Mongolia/23283/2019(H7N9)	E1	S	K	K	K	V	R	K	T	P	T	R	V	R	G	R
A/Environment/Inner Mongolia/23280/2019(H7N9)	E1	S	K	K	K	V	R	K	T	P	T	R	V	R	G	R
A/Environment/Inner Mongolia/23278/2019(H7N9)	E1	S	K	K	K	V	R	K	T	P	T	R	V	R	G	R

NA: not applicable.

^a ^Environmental samples were collected 4 days after the first specimen was obtained from the patient.

^b^ Previously reported immune escape mutation.

Residues at positions of mutations listed in Table 1 are shown as red sticks in the crystal structure of H7N9 HA (PDB: 4KOL, using YASARA). Glycosylation as seen in original structure is shown as cyan balls.

All eight H7N9 environmental virus sequences had 125T in HA, while five of eight human viruses had 125A (loss of glycosylation), indicating a human adaptation potential of this substitution [10]. The A151T mutation, which may add a new potential glycosylation motif at N149 was present in all viruses found in this report [11]. Besides, the substitution G177V was detected in HA protein, suggesting their increased affinities to human type receptors [12]. HA Q217L has been shown to be associated with increased binding to human-like α2,6 receptors. All the environment- and human-origin viruses in this study had the more avian-like Q at 217.

The amino acid 627K in PB2 protein, which has been suggested to increase virulence in mammalian models [13], occurred in all the re-emerged viruses derived from the case, but was not detected in any of the environmental viruses. No reported substitutions associated with drug resistance in the N9 protein occurred, indicating the sensitivity of the viruses to neuraminidase inhibitors [14]. However, the S31N substitution in the M2 protein indicated their resistance to adamantine [15] (Table 2). The 12 residues truncation in the C-terminus of the NS1 protein of these viruses was also observed in H7N9 sequences in 2017.

**Table 2:** List of substitutions associated with mammalian adaption, drug resistance and virulence, in viral sequences recovered from an infected patient and from environmental samples collected nearby, Inner Mongolia Autonomous region, China, April 2019

Protein	Mutation	Function	Amino acid	Number of human viruses	Number of environmental viruses
HA	A125T^a^	Introduces a glycosylation sequon at 123N and increased avian receptor specificity [10].	A	3	0
			T	3	8
			A/T	2	0
HA	G177V^a^	Increased virus binding to human-type receptors.	V	8	8
HA	Q217L^a^	Increased virus binding to human-type receptors.	Q	8	8
PB2	K526R	Enhance the 627K and 701N function.	R	8	8
PB2	M535L	M535L and K627E, restored the polymerase activity.	L	8	8
PB2	E627K	Increased virulence in mammalian models.	K	8	0
			E	0	8
PA	K356R	Host signature amino acids (avian to human adaptation resulting in increased replication and pathogenicity in mammals)	R	8	8
PA	S409N	Host signature amino acids (avian to human adaptation resulting in increased replication and pathogenicity in mammals)	N	8	8
M2	A30S	Reduced susceptibility to licensed anti-influenza medications.	S	8	8
M2	S31N	Reduced susceptibility to licensed anti-influenza medications.	N	8	8
NS1	P42S	Altered virulence in mice.	S	8	8
NS1	N205S	Altered antiviral response in host.	S	8	8
NS1	E218stop	Truncation that removes 12 residues from C-terminus with potential effect on pathogenicity [17].	Stop	8	8
NS2	T48A	Altered antiviral response in host.	A	8	8

^a^ H7 numbering.

## Discussion

Among zoonotic influenza A viruses, influenza A(H7N9) viruses have caused a large number of reported human infections. As one of the strategies for H7N9 prevention and control, vaccination with an H5/H7 bivalent influenza vaccine was adopted in poultry in mainland China since September 2017. It has been reported that vaccination has resulted in reduced isolation rate of H7N9 viruses in poultry by 93.3% [16]. No H7N9 human cases was reported since February 2018. However, in late March 2019, we identified one HPAI H7N9 human case with fatal outcome, and HPAI H7N9 viruses with high genome identity to those of the case were detected from environmental samples. Together, these HPAI H7N9 viruses formed a subclade which exhibited a long genetic distance to the previously reported HPAI H7N9 viruses (Figure 1). This suggests that H7N9 viruses might still circulate in poultry at a low level in limited locations. In addition, several immune escape mutations, which had not been detected in previously reported HPAI H7N9 viruses, occurred in the HA1 proteins of these viruses (Table 1). The antigenic features of these HPAI H7N9 viruses may differ from the current HPAI H7N9 candidate vaccine strain. Phenotypic features, including antigenic characterisations and receptor binding profiles, need to be investigated in further studies. In conclusion, the detection of this HPAI H7N9 in a human raises a concern for the virus surveillance in both human and avian species, and reminds us that there is still a long way to go to control H7N9 viruses.
